# A Comparison of Heart Pulsations Provided by Forcecardiography and Double Integration of Seismocardiogram

**DOI:** 10.3390/bioengineering9040167

**Published:** 2022-04-09

**Authors:** Emilio Andreozzi, Jessica Centracchio, Daniele Esposito, Paolo Bifulco

**Affiliations:** Department of Electrical Engineering and Information Technologies, University of Naples Federico II, Via Claudio, 21, 80125 Napoli, Italy; jessica.centracchio@unina.it (J.C.); daniele.esposito@unina.it (D.E.); paolo.bifulco@unina.it (P.B.)

**Keywords:** Forcecardiography, Seismocardiography, heart vibrations, piezoelectric sensor, accelerometer, cardiac monitoring

## Abstract

Seismocardiography (SCG) is largely regarded as the state-of-the-art technique for continuous, long-term monitoring of cardiac mechanical activity in wearable applications. SCG signals are acquired via small, lightweight accelerometers fixed on the chest. They provide timings of important cardiac events, such as heart valves openings and closures, thus allowing the estimation of cardiac time intervals of clinical relevance. Forcecardiography (FCG) is a novel technique that records the cardiac-induced vibrations of the chest wall by means of specific force sensors, which proved capable of monitoring respiration, heart sounds and infrasonic cardiac vibrations, simultaneously from a single contact point on the chest. A specific infrasonic component captures the heart walls displacements and looks very similar to the Apexcardiogram. This low-frequency component is not visible in SCG recordings, nor it can be extracted by simple filtering. In this study, a feasible way to extract this information from SCG signals is presented. The proposed approach is based on double integration of SCG. Numerical double integration is usually very prone to large errors, therefore a specific numerical procedure was devised. This procedure yields a new displacement signal (DSCG) that features a low-frequency component (LF-DSCG) very similar to that of the FCG (LF-FCG). Experimental tests were carried out using an FCG sensor and an off-the-shelf accelerometer firmly attached to each other and placed onto the precordial region. Simultaneous recordings were acquired from both sensors, together with an electrocardiogram lead (used as a reference). Quantitative morphological comparison confirmed the high similarity between LF-FCG and LF-DSCG (normalized cross-correlation index >0.9). Statistical analyses suggested that LF-DSCG, although achieving a fair sensitivity in heartbeat detection (about 90%), has not a very high consistency within the cardiac cycle, leading to inaccuracies in inter-beat intervals estimation. Future experiments with high-performance accelerometers and improved processing methods are envisioned to investigate the potential enhancement of the accuracy and reliability of the proposed method.

## 1. Introduction

The mechanical activity of the beating heart generates weak forces on the thoracic surface and the whole body. On one hand, the pulsatile force developed by the heart contraction propagates through various chest tissues and reaches the surface, thus causing small movements of the chest wall that can be felt by contact with the hands (practice known as palpation). On the other hand, the blood flowing into the great vessels generates recoil forces that result in subtle displacements of the body center of mass, mainly along the superior-inferior axis (head to feet). The quantitative, non-invasive assessment of the mechanical aspects of cardiac function dates back to the second half of the XIX century, with the seminal works of Marey [1] and Gordon [2]. Marey described a direct method to obtain a graphical representation of the vibrations induced on the animal and human chest wall by the beating heart. Gordon presented an apparatus to record the cardiac-induced whole-body vibrations, based on the registration of motions of a subject laying on a suspended bed, which appeared to be synchronous with subject’s heart beats. Since then, a number of novel methods and apparatuses were proposed to record the low frequency vibrations of the precordium and the whole body, which were based on several different physical principles [3,4]. Only a few of them gained sufficient attention to be extensively investigated as valuable tools for cardiovascular diseases discrimination, namely Apexcardiography (ACG) [5], Ballistocardiography (BCG) [6], and Seismocardiography (SCG) [7,8,9]. While ACG and BCG were originally based on displacement measurements, SCG was based on acceleration measurements. ACG and SCG were used to capture the chest wall movements, while BCG reflected the whole-body vibrations due to the center of mass displacements. However, they all had in common the use of cumbersome instrumentation and uneasiness of signals interpretation, which made them lose their appeal as practical indirect methods for the evaluation of the cardiac function, especially with the emergence of ultrasound imaging technologies [7]. In the last decade, the availability of small and lightweight accelerometers based on microelectromechanical systems (MEMS) technologies gave rise to a new research trend that largely focused on SCG [7,8,9,10], particularly to develop wearable applications for continuous, long-term monitoring of both healthy and pathological subjects. Indeed, the typical SCG signal is made of infrasonic oscillations, with various peaks and valleys that have a temporal relationship with important cardiac cycle events, such as heart valves opening and closures. From these fiducial markers, different cardiac time intervals can be obtained (e.g., inter-beat interval, isovolumic contraction time, pre-ejection period, left ventricular ejection time, isovolumic relaxation time, total filling time), which play a major role in the clinical evaluation of several cardiac pathologies [7,8,9].

Lately, Forcercardiography (FCG) has been proposed as a novel technique to measure the cardiac-induced vibrations of the chest wall via force sensors [11]. FCG has first been introduced by using sensors based on force sensing resistors (FSR), which had already been used to monitor muscle contraction [12,13,14]. These FSR-based sensors have also proved capable of capturing a respiratory-related signal, referred to as Forcerespirogram (FRG), thus standing as promising devices for cardiorespiratory monitoring [15]. Very recently, a new FCG sensor based on a piezoelectric transducer has been proposed, which captures respiration, infrasonic cardiac vibrations and heart sounds, simultaneously from a single contact point on the chest [16]. The infrasonic cardiac vibrations recorded by the FCG sensor can be divided in two main components: a large, low-frequency component, referred to as LF-FCG, that features a typical negative peak usually occurring at the end of the ECG T-wave; a high-frequency component, referred to as HF-FCG, that presents an SCG-like morphology [11,16]. Very recently, it has been shown that HF-FCG is able to provide the timings of aortic valve opening (AO) events and estimates of pre-ejection period with high accuracy and precision as compared to SCG [17]. The LF-FCG component seems to be related to ventricular emptying and filling, which cannot be appreciated directly in SCG recordings, nor extracted by simple low-pass filtering [11]. The reason could lie in the inherent poor sensitivity that accelerometers exhibit to slow movements. Undoubtedly, the low-frequency displacements associated to the ventricular volume variations usually result in extremely smaller accelerations as compared to those produced by heart valves snaps, thus making their observation in SCG recordings impractical. To the best of our knowledge, no successful approaches have been proposed yet in literature to recover information on ventricular emptying and filling from SCG.

In this study, we present a method to extract this information, which is based on numerical double integration of accelerometric SCG signals. In fact, we show that by integrating an SCG signal two times via a proper numerical procedure, a low-frequency displacement signal can be obtained, which results very similar to the LF-FCG signal. To this aim, signals from a piezoelectric FCG sensor [16] and a MEMS accelerometer were synchronously acquired from the same location onto the chest wall, together with an ECG lead that provided a reliable reference for the heartbeats. The similarity of the low-frequency displacement signal obtained from double integration of SCG with the LF-FCG was assessed via different normalized cross-correlation (NCC) indices. Moreover, the consistency of the new displacement signal within the cardiac cycle was assessed by carrying out statistical analyses on the heartbeats detection performances, as well as on the accuracy and precision of the related inter-beat intervals estimations.

## 2. Materials and Methods

### 2.1. FCG and SCG Sensors

This article presents a retrospective analysis of signals acquired during the study described in [16]. The materials and methodologies adopted for the experiments are reported below, but no measurements were carried out during this study. FCG signals were acquired via the piezoelectric FCG sensor and the related conditioning circuit described in [16]. The sensor is composed by a lead-zirconate-titanate piezoelectric disk, with a diameter of 30.50 mm and an electrical capacitance of 22 nF (measured at 2 kHz via a GWINSTEK LCR-816 LCR meter, Good Will Instrument Co., Ltd., No.7-1, Jhongsing Road., Tucheng Dist., New Taipei City 236, Taiwan), equipped with a dome-shaped mechanical coupler that ensures a good mechanical transduction from subjects’ skin [11,15,16]. The dorso-ventral SCG signals were simultaneously acquired by recording the *z*-axis acceleration signals of a Freescale MMA7361 accelerometer, which was fixed onto the FCG sensor (as in [11,16]).

### 2.2. Measurement Setup and Protocol

**Figure 1:**
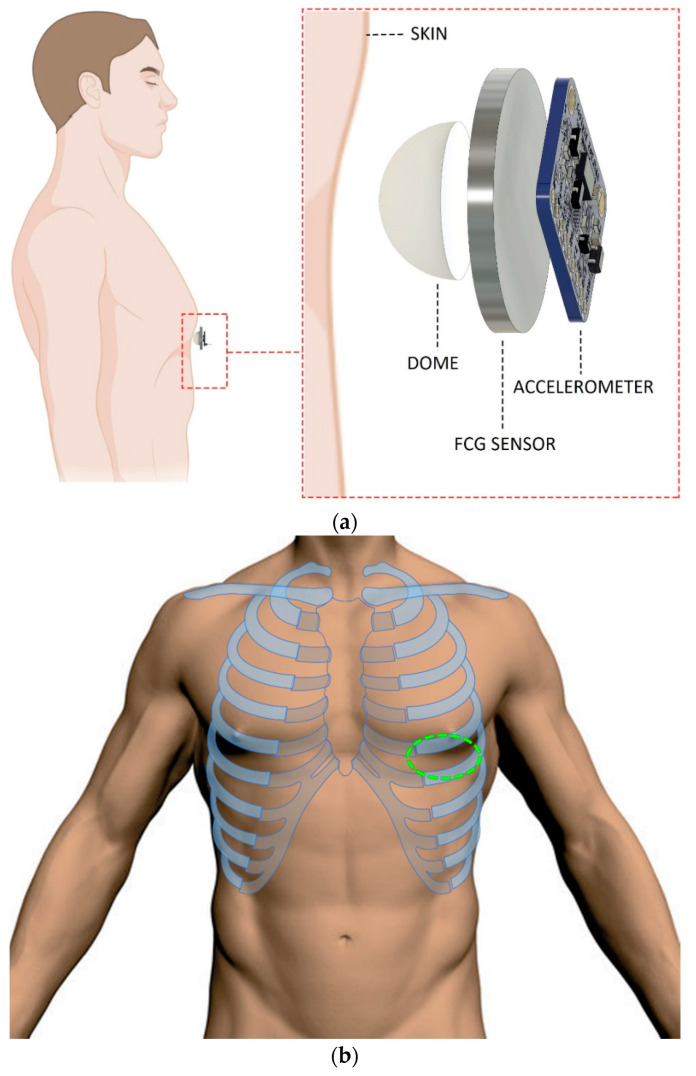
(**a**) Lateral view of sensors assembly applied on the chest of a subject; (**b**) frontal view of sensors positioning area on the chest (green dashed line).

The FCG and SCG sensors assembly was placed onto the chest of each subject via a medical adhesive tape, by roughly locating the point of maximal impulse (i.e., the maximum signal amplitude point around the fifth intercostal space on the midclavicular line), and then fastened with a belt around the thorax (Figure 1). Simultaneous acquisitions of FCG and SCG signals, together with an ECG lead I provided by a WelchAllyn Propaq^®^ Encore monitor (Welch Allyn Inc., New York, NY, USA), were carried out via a National Instrument NI-USB4431 DAQ board (National Instruments Corp., 11500 N Mopac Expwy, Austin, TX 78759-3504, USA), with 24-bit precision and 10 kHz sampling frequency.

Five healthy volunteers (3 males, 2 females, age 36.6 ± 11.0), who signed a written informed consent, were asked to comfortably sit on a chair, leaning against the seatback while keeping their back straight. Multiple acquisitions were performed for each subject in two respiratory conditions, i.e., quiet breathing and apnea.

### 2.3. Signals Processing

The signals acquired during quiet breathing and apneas were processed and analyzed separately. All processing and analyses were carried out in MATLAB^®^ R2017b (The MathWorks, Inc., 1 Apple Hill Drive, Natick, MA 01760, USA).

#### 2.3.1. FCG Sensor Signals Processing

As shown in [15,16], the raw FCG sensor signals contain information on both respiratory and cardiac activity, referred to as Forcerespirogram (FRG) and Forcecardiogram (FCG), respectively. In signals acquired during quiet breathing, the FRG was first extracted via a 3rd order Savitzki-Golay filter [18], with a frame length corresponding to about a 1.5 s interval. Then, the FRG thus obtained was subtracted from the raw FCG sensor signal to isolate the actual FCG signal, which was further processed via a 2nd order Butterworth band-pass filter with cut-off frequencies set at 0.5–5 Hz, to eventually obtain the LF-FCG signal. In signals acquired during apneas, the FRG subtraction step was not carried out since no respiratory activity had been captured, so the raw FCG sensor signal was directly band-pass filtered in the 0.5–5 Hz frequency band to extract the LF-FCG signal.

#### 2.3.2. SCG Signals Processing

Numerical double integration of SCG signals was performed via the following procedure. Before the integration, DC removal was performed on the accelerometric signal by subtracting its time average. The actual numerical integrations were then performed via the MATLAB^®^ fa punction “*cumtrapz*”, which computes an approximation of the cumulative integral of a signal via the trapezoidal method. After each integration step, the resulting signal was iteratively processed via a 4th order, zero-lag, Butterworth high-pass filter with 0.6 Hz cut-off frequency for 40 times. This strong high-pass filtering is a fundamental step, since it allows filtering out spurious low-frequency components produced by the numerical integration, which show up with extremely higher amplitudes as compared to the signals of interest. Finally, the double-integrated signal, referred to as “Displacement SCG” (DSCG), was processed via the same band-pass filter used to extract the LF-FCG, so as to obtain the low-frequency component of the DSCG, referred to as LF-DSCG.

### 2.4. Morphological Comparison

The morphologies of LF-FCG and LF-DSCG signals were compared by evaluating the following NCC indices: (a) between the whole signals; (b) between single corresponding heart beats extracted from the two signals; (c) between the ECG-triggered ensemble averages (synchronized with R-peaks) of the two signals. This analysis was carried out separately for signals acquired during quiet breathing and during apneas.

### 2.5. Statistical Analyses

The consistency of the LF-DSCG signal within the cardiac cycle was assessed by evaluating its ability to detect the heartbeats, as well as the accuracy and precision of the derived heart rate measurements. These performances were assessed by assuming the ECG signal as the reference, and were further compared with those achieved by the LF-FCG signal. To this aim, the R-peaks were first located in the ECG signal via the “BioSigKit” MATLAB^®^ toolbox, which implements the well-known Pan and Thompkins algorithm [19]. Then, the heartbeats were detected both in the LF-FCG and LF-DSCG signals by considering the negative peaks of the first derivatives of the signals as fiducial markers, which were located by taking advantage of the *a priori* knowledge of R-peaks locations [16]. The annotation of the missed heartbeats in the LF-FCG and LF-DSCG signals was carried out by comparison with the ECG. Finally, the inter-beat intervals computed from the fiducial markers of the LF-FCG and LF-DSCG signals were compared with those obtained from the ECG R-peaks via regression, correlation and Bland-Altman analyses, which were carried out by using the MATLAB^®^ function “bland-altman-and-correlation-plot” [20]. The intervals related to the missed heartbeats were excluded from these statistical analyses.

## 3. Results

### 3.1. Morphological Comparison of LF-FCG and LF-DSCG Signals

#### 3.1.1. Apnea

**Figure 2:**
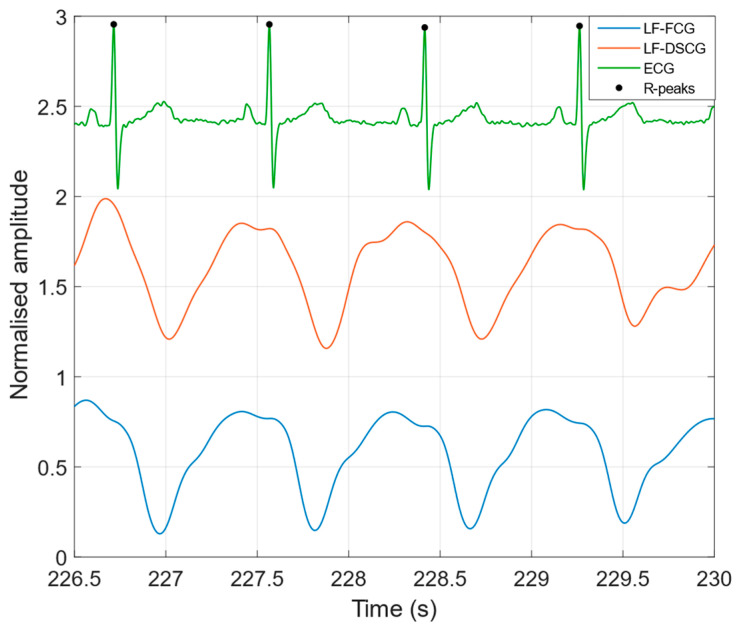
LF-FCG, LF-DSCG and ECG signals acquired during apneas (subject #2).

Figure 2 shows a comparison of the LF-FCG and LF-DSCG signals acquired during apneas, along with the ECG signal acquired concurrently. In Table 1 the NCC indices of the LF-FCG and LF-DSCG signals acquired during apneas are reported for each subject. For the indices related to the whole signals (NCC_W_) and to the ECG-triggered ensemble averages (NCC_E_), a single value was reported, while for the indices related to single heartbeats, the mean and the standard deviation (SD) of the indices scored for all heartbeats were reported for each subject, and referred to as NCC_MEAN_ and NCC_SD_, respectively. The ECG-triggered ensemble averages scored NCC_E_ of 0.93 ± 0.054 (mean ± SD), which turned out to be in excess of 0.94 for all but one subject (#5). On single heartbeats, LF-FCG and LF-DSCG scored NCC_MEAN_ of 0.88 ± 0.083, which turned out to be in excess of 0.9 for 3 out of 5 subjects and reduced by less than 5% with respect to NCC_E_ in all but one subject (#1), and NCC_SD_ of 0.063 ± 0.057, which turned out to be lower than 0.1 for all but one subject (#1). The whole LF-FCG and LF-DSCG signals scored NCC_W_ of 0.82 ± 0.15 and turned out to be reduced by less than 15% with respect to the related NCC_MEAN_ and NCC_E_ in all but one subject (#1).

#### 3.1.2. Quiet Breathing

**Figure 3:**
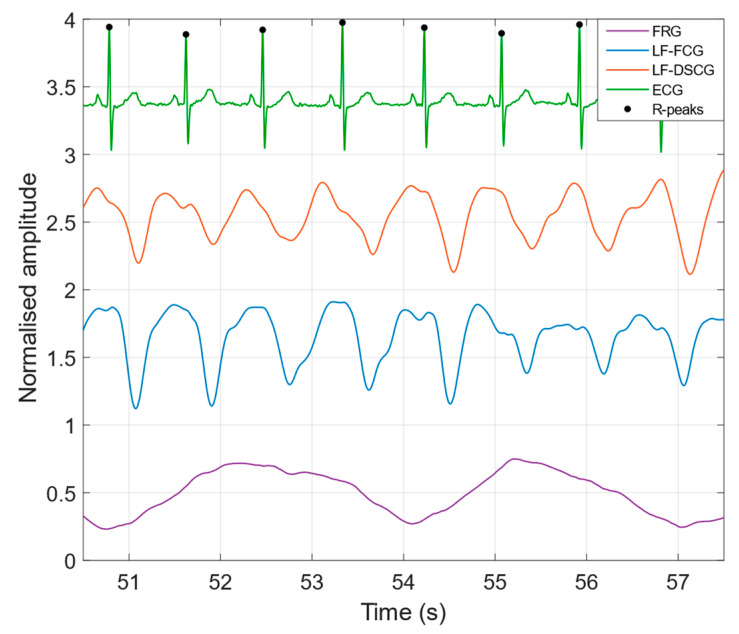
FRG, LF-FCG, LF-DSCG and ECG signals acquired during quiet breathing (subject #2).

Figure 3 shows a comparison of the LF-FCG and LF-DSCG signals acquired during quiet breathing, along with the ECG signal acquired simultaneously and the FRG signal (respiratory activity) extracted from the raw FCG sensor signal. In Table 2 the NCC indices of the LF-FCG and LF-DSCG signals acquired during quiet breathing are reported for each subject. The ECG-triggered ensemble averages scored NCC of 0.90 ± 0.027, which turned out to be in excess of 0.90 for 3 out of 5 subjects. On single heartbeats, LF-FCG and LF-DSCG scored NCC_MEAN_ of 0.81 ± 0.022, which turned out to be in excess of 0.8 for 3 out of 5 subjects and reduced by about 10% with respect to the related NCC_E_ for all subjects, and NCC_SD_ of 0.12 ± 0.011, which turned out to be lower than 0.15 for all subjects. The whole LF-FCG and LF-DSCG signals scored NCC of 0.72 ± 0.12 and turned out to be reduced by less than 10% with respect to the related NCC_MEAN_ and less than 20% with respect to the related NCC of the ensemble averages, in all but one subject (#5).

### 3.2. Statistical Analyses

#### 3.2.1. Apnea

**Figure 4:**
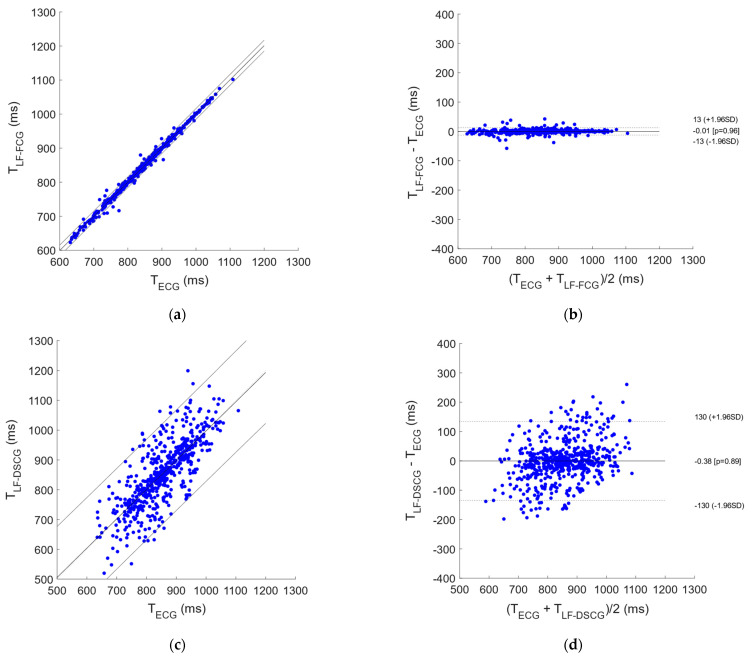
Statistical analyses on inter-beat intervals extracted from LF-FCG and LF-DSCG signals during apneas. (**a**) Results of regression and correlation analyses of LF-FCG vs. ECG; (**b**) results of Bland-Altman analysis of LF-FCG vs. ECG; (**c**) results of regression and correlation analyses of LF-DSCG vs. ECG; (**d**) results of Bland-Altman analysis of LF-DSCG vs. ECG.

In the signals acquired from all subjects during apneas, 4 and 63 missed heartbeats were found, respectively, in LF-FCG and LF-DSCG, out of a total of 698 heartbeats detected in the simultaneously acquired ECG signals. Consequently, LF-FCG and LF-DSCG scored a sensitivity of 99.4% and 91.0% respectively. Figure 4 shows the results of the regression, correlation and Bland-Altman analyses that were performed on the inter-beat intervals extracted from LF-FCG and LF-DSCG, as compared to those extracted from ECG. The inter-beat intervals related to the missed heartbeats were discarded from the analyses, which were performed on a total of 670 intervals for LF-FCG and 581 intervals for LF-DSCG. The statistical analyses reported, for LF-FCG, a slope and intercept of 1.003 and −2.8 ms (R^2^ = 0.995) and a non-significant bias (*p* = 0.96) with limits of agreement of ±12.6 ms; for LF-DSCG, a slope and intercept of 0.983 and 14.1 ms (R^2^ = 0.603) and a non-significant bias (*p* = 0.89) with limits of agreement of ±134.4 ms.

#### 3.2.2. Quiet Breathing

**Figure 5:**
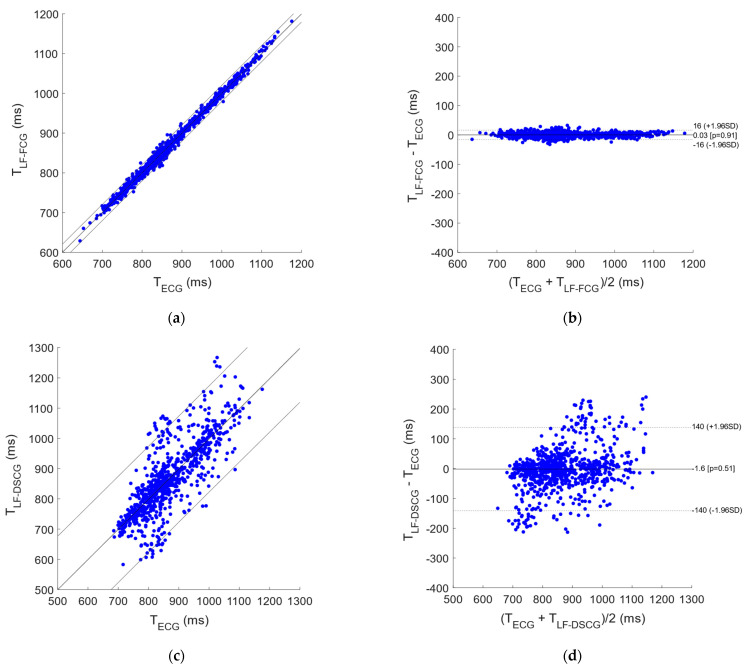
Statistical analyses on inter-beat intervals extracted from LF-FCG and LF-DSCG signals during respiration. (**a**) Results of regression and correlation analyses of LF-FCG vs. ECG; (**b**) results of Bland-Altman analysis of LF-FCG vs. ECG; (**c**) results of regression and correlation analyses of LF-DSCG vs. ECG; (**d**) results of Bland-Altman analysis of LF-DSCG vs. ECG.

A total of 1026 heartbeats were detected in the ECG signals acquired from all subjects during quiet breathing. In the related LF-FCG signals a total of 11 missed heartbeats were found, while 125 missed heartbeats were found in the LF-DSCG signals. Consequently, LF-FCG and LF-DSCG scored a sensitivity of 98.9% and 87.8%, respectively. Figure 5 shows the results of the statistical analyses that were performed on the inter-beat intervals extracted from the signals acquired during quiet respiration. The inter-beat intervals related to the missed heartbeats were discarded from the analyses, which were performed on a total of 1000 intervals for LF-FCG and 819 intervals for LF-DSCG. Slope and intercept of 0.9985 and 1.4 ms (R^2^ = 0.993) were found for LF-FCG, as well as a non-significant bias (*p* = 0.91) with limits of agreement of ±16.2 ms, while for LF-DSCG, a slope and intercept of 0.997 and 1.3 ms (R^2^ = 0.631) and a non-significant bias (*p* = 0.51) with limits of agreement of ±139.9 ms were found.

## 4. Discussion

This study focused on a method to extract information on ventricular emptying and filling events from the SCG signal. SCG analysis usually discards low-frequency vibrations because slow motions result in extremely small accelerations. To the best of our knowledge, no successful approaches have been proposed yet in literature to recover this information from SCG. To address this issue, a specific numerical procedure based on double integration of SCG was presented. The proposed method yields a new displacement signal (DSCG), which is not directly visible in SCG recordings, nor can it be extracted by simple filtering operations, also overcoming the well-known problems associated with numerical double integration (usually prone to large errors). The DSCG signal features a low-frequency component (LF-DSCG) that captures Apexcardiography-like information on heart walls displacements related to ventricular volume variations. This component also shows high similarity to the LF-FCG signal. Therefore, the morphologies of LF-DSCG and LF-FCG signals were quantitatively compared by evaluating different normalized cross-correlation indices. The ECG-triggered ensemble averages of LF-FCG and LF-DSCG scored, on average, NCC indices in excess of 0.9, both during apnea and quiet breathing. These results show that the proposed approach based on numerical double integration of SCG signals successfully recovered the essential features of the LF-FCG morphology, i.e., those that got through the ensemble averaging operation. The morphological comparison of single heartbeats (without ensemble averaging) reported slightly lower correlations (on average 0.8 < NCC_MEAN_ < 0.9), which turned out to be reduced by less than 5% in all but one subject during apneas, and by about 10% in all subjects during quiet breathing. These findings suggest that the LF-DSCG could be able to monitor morphological variations in different heartbeats. However, the higher NCC_SD_ obtained in quiet breathing with respect to apneas suggest that the interference of the respiratory activity may impair the quality of LF-DSCG signals. Concerning the results obtained for the whole signals, while during apneas the average NCC_W_ turned out to be still higher than 0.8, during quiet breathing it reduced to about 0.7, which is by more than 10% with respect to NCC_MEAN_ and more than 20% with respect to NCC_E_. This result implies that LF-DSCG, as obtained via the proposed approach, is not able to track the beat-by-beat changes in morphology and amplitude of LF-FCG with reasonable accuracy.

Moreover, statistical analyses were carried out on the heartbeats detection performances, as well as on the accuracy and precision of the related inter-beat intervals estimations, to assess the consistency of the LF-DSCG within the cardiac cycle. The results show that LF-DSCG achieved fair sensitivity in heart beats detection (about 90%) but exhibited moderate consistency within the cardiac cycle, which led to limits of agreement with ECG higher than 130 milliseconds for inter-beat interval estimation.

In conclusion, when properly processed with a specific numerical procedure, the SCG signal could provide information on heart walls displacements, as the FCG. This information was first captured by Apexcardiography, which has been shown to give important insights into the mechanical behavior of the beating heart, both under physiological and pathological conditions [21,22,23,24,25,26,27,28]. This technique has lost its appeal due to the cumbersome instrumentation required and to the clear superiority of Echocardiography as a diagnostic tool in clinical settings. Nonetheless, FCG and DSCG stand as promising ways of recovering the wide knowledge acquired on ACG, both in normal and pathological conditions, and transfer it to wearable monitoring applications. The results of this study show that LF-FCG exhibits higher consistency within the cardiac cycle with respect to LF-DSCG. This suggests that FCG currently outperforms the double integration of SCG. However, further experiments with high-performance accelerometers and advanced processing methods could improve the accuracy and reliability of DSCG [29]. Finally, a deeper investigation through the comparison with Echocardiography is envisioned in the future to assess the relationship of FCG and DSCG with actual heart walls displacements. To this aim, the application of the proposed double integration procedure to tridimensional SCG signals will also be investigated, as it has been shown that 3D accelerations may bring more comprehensive information about cardiac-induced vibrations of the chest wall [30].

## Figures and Tables

**Table 1:** Normalized cross-correlation indices of the LF-FCG and LF-DSCG signals acquired during apneas. The correlation indices were computed between the whole signals, between single corresponding heartbeats (mean and SD of correlation indices are reported) and between the ECG-triggered ensemble averages.

Subject	Whole Signals	Single Heartbeats		Ensemble Averages
		Mean	SD	
#1	0.6003	0.7782	0.1574	0.9647
#2	0.8961	0.9132	0.04177	0.9470
#3	0.9315	0.9423	0.02863	0.9507
#4	0.9473	0.9496	0.01551	0.9556
#5	0.7300	0.7932	0.07253	0.8341

**Table 2:** Normalized cross-correlation indices between LF-FCG and LF-DSCG signals acquired during quiet breathing. The correlation indices were computed between the whole signals, between single corresponding heartbeats (mean and SD of correlation indices are reported) and between the ECG-triggered ensemble averages.

Subject	Whole Signals	Single Heartbeats		Ensemble Averages
		Mean	SD	
#1	0.7125	0.7919	0.1232	0.8728
#2	0.7840	0.8248	0.1085	0.9262
#3	0.8060	0.8244	0.1195	0.9251
#4	0.7649	0.8145	0.1040	0.9012
#5	0.5166	0.7739	0.1306	0.8728

## Data Availability

Data are available upon reasonable request.
